# Evaluation of Anti-Inflammatory Activity of *Citrus latifolia* Tanaka Essential Oil and Limonene in Experimental Mouse Models

**DOI:** 10.1155/2013/859083

**Published:** 2013-05-15

**Authors:** Raquel Kummer, Fernanda Carolina Fachini-Queiroz, Camila Fernanda Estevão-Silva, Renata Grespan, Expedito Leite Silva, Ciomar Aparecida Bersani-Amado, Roberto Kenji Nakamura Cuman

**Affiliations:** ^1^Department of Pharmacology and Therapeutics, State University of Maringá, 870020-900 Maringá, PR, Brazil; ^2^Department of Chemistry, State University of Maringá, 870020-900 Maringá, PR, Brazil

## Abstract

The genus *Citrus* (Rutaceae) includes several species of plants that produce some of the most cultivated fruits in the world, providing an appreciable content of essential oil. In folk medicine, they are used as a cholagogue, antipyretic, anti-inflammatory, sedative, and antitoxic effects. Lemon essential oil has been used since ancient times for its antiseptic, carminative, diuretic, and eupeptic effects. In this study, we investigated the anti-inflammatory activity of *Citrus latifolia * Tanaka essential oil (CLEO) and its main constituent LIM. In the cell viability assay, CLEO and LIM (3, 10, 30, and 90 **μ**g/mL) had low cytotoxicity. In zymosan-induced peritonitis, LIM (500 mg/kg) decreased the infiltration of peritoneal exudate leukocytes and decreased the number of polymorphonuclear leukocytes. *In vitro* chemotaxis revealed that CLEO and LIM (1, 3, and 10 *µ*g/mL) promoted a significant reduction of neutrophil migration toward fMLP and LTB_4_. LIM (500 mg/kg) also reduced TNF-**α** levels but did not alter IL-10 levels in the peritoneal exudate. In conclusion, this study showed that LIM isolated from CLEO had potential anti-inflammatory effects, likely by inhibiting proinflammatory mediators present in inflammatory exudate and leukocyte chemotaxis.

## 1. Introduction

The genus *Citrus* (Rutaceae) includes several species of plants that produce some of the most cultivated fruits in the world, including oranges and lemons, which have an appreciable content of essential oil. In folk medicine, they are used as a cholagogue and for their digestive, tonic, antipyretic, anti-inflammatory, sedative, and antitoxic effects [1–4]. Essential oils of plants from the genus *Citrus* have monoterpenes and sesquiterpenes as their constituents [5, 6]. The literature indicates the presence of 50 or more different compounds obtained from citrus peel, whereas limonene (LIM) is the main compound [3, 7]. *Citrus latifolia* Tanaka is popularly known as Tahiti lime and grows well in tropical regions [8]. 

Lemon essential oil is a complex mixture of LIM, *γ*-terpinene, citral, linalool, and *β*-caryophyllene, among others [9]. Since ancient times, it has been used for its antiseptic, carminative, diuretic, and eupeptic effects [2]. Some of its compounds, including *β*-caryophyllene, LIM, and linalool, have anti-inflammatory effects [10–12]; *α*-pinene and *β*-pinene inhibit the synthesis of nitric oxide (NO), suggesting an antioxidant effect [3], and recent reports showed that *β-*pinene exerts an antispasmodic effect on the rat ileum and provokes antinociceptive actions [13].

LIM is one of the most common terpenes in nature and has been used as a flavoring agent in common food items, such as fruit juices, soft drinks, and ice cream, and in the cosmetics and pesticide industries [11, 14]. LIM has been shown to exert antiulcerogenic, gastroprotective, chemopreventive, antiproliferative, insecticide, antimicrobial, and immunomodulatory effects [15–18]. This compound has also been shown to have anti-inflammatory effects by reducing eosinophil chemotaxis and MCP-1 production [11]. It effectively inhibited lipopolysaccharide- (LPS-) induced NO and prostaglandin E_2_ (PGE_2_) production in macrophages [19] and decreased interleukin-1*α* (IL-1*α*) levels in normal human undifferentiated NCTC 2544 keratinocytes [20].

The biological activity of extracts of herbs has been widely studied, but few studies have evaluated the effects of essential oils obtained from plants of the genus *Citrus* and its constituents on anti-inflammatory activity. The present study investigated the anti-inflammatory activity of *Citrus latifolia* Tanaka essential oil (CLEO) and its main constituent, LIM.

## 2. Materials and Methods

### 2.1. Plant Material and Extraction of Essential Oil

The fruits of *Citrus latifolia* Tanaka were commercially purchased in Maringá, PR, Brazil. The essential oils were extracted from the flavedos of fruits of *Citrus latifolia* Tanaka (690 g) by conventional steam distillation using a Clevenger-type apparatus for 2 h. The obtained essential oil was dried over sodium sulfate and stored at 4°C in dark vials until tested. The yield of CLEO was 2.76% v/w. The constituent limonene was isolated from CLEO as fractions of hydrodistillated oil.

### 2.2. Analysis of the Essential Oil and Compound Identification

#### 2.2.1. Gas Chromatography-Mass Spectrometry

 Gas chromatography was performed with a Thermo Electron Corporation Focus GC model under the following conditions: DB-5 capillary column (30 m × 0.32 mm, 0.50 mm), column temperature (60°C for 1 min to 180°C at 3°C/min), injector temperature (220°C), detector temperature (220°C), split ratio (1 : 10), carrier gas (He), and flow rate (1.0 mL/min). The volume injected (1 *μ*L) was diluted in chloroform (1 : 10). The GC-MS analysis was performed in a Quadrupole mass spectrometer (DSQ II model, Thermo Electron Corporation) that operated at 70 V. The identification of the individual compounds was based on comparisons of their GC retention indices on an apolar column and comparisons with mass spectra of authentic standards purchased from Sigma-Aldrich [21].

#### 2.2.2. Nuclear Magnetic Resonance

Nuclear magnetic resonance (NMR) was used to prove the chemical structure of the essential oil constituents identified by GC-MS.  ^13^C NMR (75.45 MHz) spectra were recorded in a deuterated chloroform (CDCl_3_) solution using a Mercury-300BB spectrometer, with *δ* (ppm) and spectra referenced to CDCl_3_ (*δ* 77.00 for  ^13^C) as an internal standard.

### 2.3. Animals

For the evaluation of anti-inflammatory effects, male BALB/c mice were used (20–25 g). The animals were obtained from the Central Animal House of the State University of Maringá. The animals were housed at 22 ± 2°C under a 12 h/12 h light/dark cycle. The experimental protocols were approved by the Ethical Committee in Animal Experimentation of the State University of Maringá (CEAE/UEM066/2010).

### 2.4. Bioassays for Cytotoxic Activity

The MTT (3-[4,5-dimethylthiazol-2-yl]-2,5-diphenyl-2H-tetrazolium bromide) assay is based on the mitochondrial enzyme reduction of tetrazolium dye that detects and determines cell viability [22]. The neutrophils were obtained from the peritoneal cavity of BALB/c mice 4 h after zymosan injection (1 mg/cavity, i.p.). Briefly, the cells (5 × 10^5^ cells/well) were exposed to CLEO (3, 10, 30, and 90 *μ*g/mL) or LIM (3, 10, 30, and 90 *μ*g/mL) for 90 min at 37°C in 5% CO_2_. CLEO and LIM emulsions were prepared with RPMI medium and emulsified by sonication. A volume of 10 *μ*L MTT (5 mg/mL; Sigma) was added to each well. After 2 h, 150 *μ*L of the supernatant was removed, and 100 *μ*L of dimethyl sulfoxide was added to each well. The cells were incubated at 25°C for an additional 10 min, and absorbance was measured using a Biochrom Asys Expert plus microplate reader (Asys) at a wavelength of 540 nm. The values of the blank wells were subtracted from each well of treated and control cells. The percentage of viability was determined by the following formula:

(1) %  Viable  cells:Absorbance  of  the  treated  cells−Absorbance  of  the  blankAbsorbance  of  the  control−Absorbance  of  the  blank×100

### 2.5. Anti-Inflammatory Activity

#### 2.5.1. *In Vitro* Chemotaxis Assay

To evaluate the effects of CLEO and LIM on chemotaxis, neutrophils were obtained from the peritoneal cavity of BALB/c mice 4 h after the zymosan injection (1 mg/cavity, i.p). The cell number was adjusted to 1 × 10^6^ cells/mL in RPMI medium that contained 0.1% bovine serum albumin (BSA). The chemotaxis assay was performed using a 48-well microchemotaxis plate (Neuro Probe), in which the chambers were separated by a polyvinylpyrrolidone-free polycarbonate membrane (5 *μ*m pore size). The chemoattractants *N*-formyl methionyl leucyl phenylalanine (fMLP; 10^−6 ^M) and LTB_4_ (10^−8 ^M) and a negative control (RPMI 1640) were placed in the lower chamber. A neutrophil suspension (1 × 10^6^ cells/mL) pretreated with CLEO (1, 3, or 10 *μ*g/mL) and LIM (1, 3, or 10 *μ*g/mL) for 30 min was then placed in the upper chamber. CLEO and LIM emulsions were prepared with RPMI and emulsified by sonication. The cells were allowed to migrate into the membrane for 1 h at 37°C in 5% CO_2_. Following incubation, the membrane was washed and stained using Instant Prov (Newprove). The membrane area of each well was scored using light microscopy to count the intact cells present in five random fields. The results are expressed as the mean number of neutrophils per field and representative of three separate experiments.

#### 2.5.2. Zymosan-Induced Peritonitis in Mice

*In vivo *neutrophil migration was performed in BALB/c mice. Mice were pretreated with LIM (125, 250, or 500 mg/kg, p.o.) or 0.2% of an aqueous Tween 80 solution (0.1 mL, p.o.) as the control. Thirty minutes later, all of the animals received an intraperitoneal zymosan injection (1 mg/cavity) or an equivalent volume of vehicle (saline). Six hours after the animals were sacrificed, the cells present in the peritoneal cavity were harvested by introducing 2.0 mL of phosphate-buffered saline (PBS) that contained ethylenediaminetetraacetic acid (EDTA). Counts were then performed in total and differential cells. The results are expressed as the number of neutrophils.

#### 2.5.3. Measurements of Cytokine Levels by Enzyme-Linked Immunosorbent Assay

The levels of TNF-*α* and IL-10 were determined in peritoneal exudate in BALB/c mice. The group of mice was pretreated with LIM (500 mg/kg, p.o.) or 0.2% of an aqueous Tween 80 solution (0.1 mL, p.o.) as the control. Thirty minutes later, all of the animals received an intraperitoneal zymosan injection (1 mg/cavity) or an equivalent volume of vehicle (saline). Six hours after the animals were sacrificed, the exudate present in the peritoneal cavity was harvested by introducing 1.0 mL of PBS that contained EDTA. The samples were centrifuged at 1000 rotations per minute for 10 min at 4°C. The supernatant was separated for dosing and rapidly frozen and stored at −70°C for later analysis. We used commercial kits for the enzyme-linked immunosorbent assay according to the manufacturer's recommendations (R&D Systems, Cayman Chemical).

### 2.6. Statistical Analysis

The data are expressed as the mean ± SEM for each group. The data were statistically analyzed using one-way analysis of variance followed by Tukey's test and Student's *t*-test. Differences were considered significant at *P* < 0.05.

## 3. Results and Discussion

**Figure 1:**
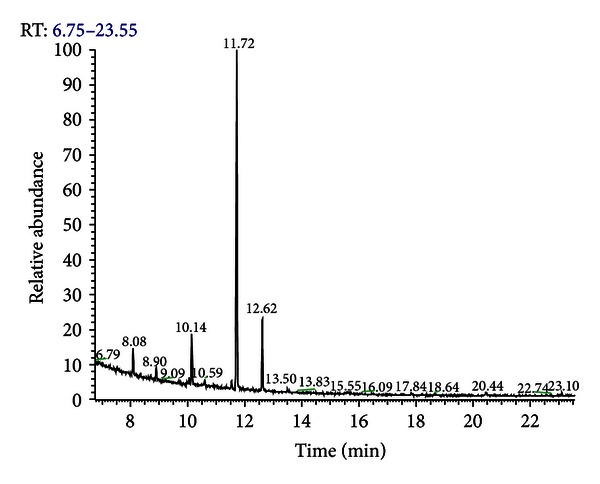
GC chromatogram of *Citrus latifolia* Tanaka essential oil. Percentual data were obtained by gas chromatography-mass spectrometry (GC-MS). Peak identification is reported in Table 1.

**Figure 2:**
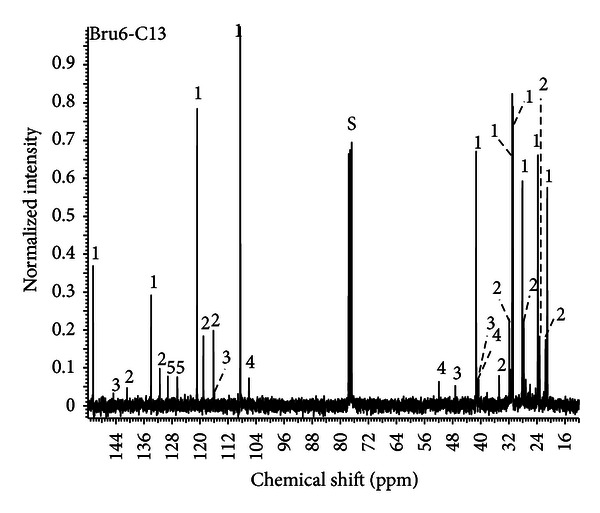
^13^C NMR spectra of the *Citrus latifolia* Tanaka essential oil in deuterated chloroform (CDCl_3_). The numbers on the peaks are attributed to majority compounds: (1) Limonene, (2) **γ**-terpinene, (3) **β**-pinene, (4) **α**-pinene, (5) *p*-cymene. S = solvent chloroform (CHCl_3_).

The chemical composition of CLEO was investigated by gas chromatography-mass spectrometry (GC-MS) and nuclear magnetic resonance (NMR). The results of the GC-MS analysis (Figure 1) showed a predominance of LIM (62%), *γ*-terpinene (14.2%), *β*-pinene (12.2%), *α*-pinene (2.8%), and *p*-cymene (1.8%), similar to previous studies [3, 6, 7]. A complete list of the components and their relative abundances is presented in Table 1. To confirm the structure of the main compounds, CLEO was studied by  ^13^C NMR (Figure 2). The chemical shift of each carbon in the experimental spectrum was compared with shifts of the spectra of pure compounds. 

In the cell viability assay, the treatments were tested at different concentrations. CLEO at concentrations of 3, 10, 30, and 90 *μ*g/mL showed cell viability of 85%, 79%, 75%, and 77%, respectively. LIM at concentrations of 3, 10, 30, and 90 *μ*g/mL showed cell viability of 88%, 78%, 77%, and 79%, respectively. Our data indicated that the CLEO and LIM treatments did not present *in vitro* cytotoxicity at any of the concentrations tested, with viability >75% at a concentration of 10 *μ*g/mL, similar to a cytotoxicity study of plants from the genus *Citrus* [3, 14].

**Figure 3:**
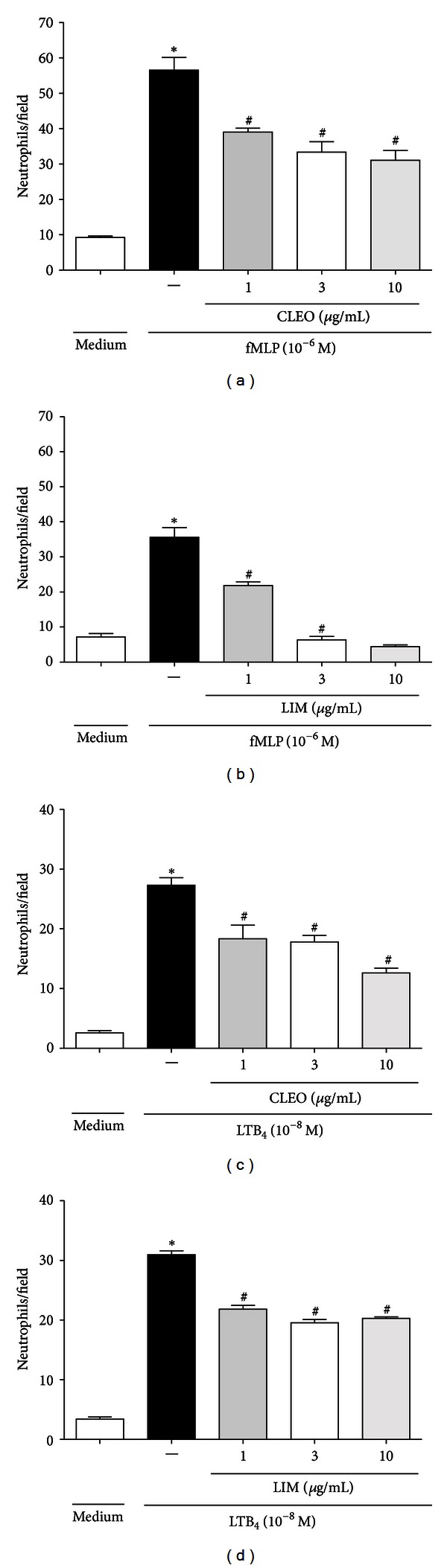
Effect of CLEO and LIM on neutrophils chemotaxis *in vitro*. Neutrophils were obtained from zymosan-induced peritonitis (1 mg/cavity,) and stimulated with fMLP (10^−6^) or LTB_4_ (10^−8^) after 30 min of treatment with CLEO (a, c) or LIM (b, d) at doses of 1, 3, and 10 **μ**g/mL. Values are mean ± S.E.M. (*n* = 5) and are representative of three independent experiments. *P < 0.05 versus medium (RPMI 1640). ^#^ *P* < 0.05 versus group of neutrophils stimulated with fMLP or LTB_4_. (One-way ANOVA, Tukey's test).

Neutrophils are first responders in an organism's rapid assault on infectious pathogens [23]. A recent study suggested that signals that arise from formyl-Met-Leu-Phe (fMLP) may predominate in directing the responses of neutrophils that have migrated to the final site of an infection [24]. The formyl peptide fMLP is a bacterial product that is recognized by neutrophils upon binding to its heterotrimeric G protein-coupled receptor, initiating signaling cascades that activate multiple pathways. These pathways include the mitogen-activated protein kinase (MAPK) and phosphatidylinositol 3-kinase (PI-3K) cascades, which are important for the development of the functional responses of neutrophils in inflammation [23, 25]. To evaluate the direct effects of CLEO and LIM on *in vitro* neutrophil chemotaxis, different concentrations were applied. The chemoattractants fMLP (10^−6 ^M) and leukotriene B_4_ (LTB_4_; 10^−8 ^M) were used. CLEO at doses of 1, 3, and 10 *μ*g/mL significantly reduced (*P* < 0.05) neutrophil migration in response to fMLP stimulation (31.32%, 40.85%, and 45.45%, resp.). LIM treatment at the same doses significantly reduced (*P* < 0.05) neutrophil migration in response to fMLP stimulation (38.68%, 82.14%, and 87.63%, resp.; Figures 3(a) and 3(b)).

LTB_4_ is a potent chemoattractant derived from arachidonic acid. It modulates diverse functions in living systems (e.g., it induces chemotaxis) [26, 27]. CLEO at doses of 1, 3, and 10 *μ*g/mL significantly reduced (*P* < 0.05) neutrophil migration in response to LTB_4_ stimulation (32.86%, 34.80%, and 54.84%, resp.). LIM treatment at the same doses also significantly reduced (*P* < 0.05) neutrophil migration in response to LTB_4_ stimulation (29.48%, 36.82%, and 34.52%, resp.; Figures 3(c) and 3(d)). fMLP and LTB_4_ were used as chemotaxic agents in the *in vitro* tests, and CLEO and LIM inhibited neutrophil migration. Our results indicate that prostanoids and cytokines are involved in this process, in which CLEO and LIM did not affect neutrophil viability at the concentrations tested, suggesting that the direct effects of the treatments on the inhibition of neutrophil chemotaxis did not occur because of toxic effects that induced cell death.

Recent studies support these results. Lemon essential oil inhibited the activity of 5-lipoxygenase (5-LOX), and the inhibitory effect of LIM was observed in eotaxin-induced eosinophil chemotaxis [11, 28]. Neutrophils have many cell surface receptors that are coupled to PI3K-dependent processes, including chemotaxis receptors [29]. In an under-agarose assay, neutrophils predominantly migrated toward the fMLP chemoattractant via p38 MAPK, whereas LTB_4_-induced migration (i.e., an intermediary chemoattractant) was PI3K dependent [24, 30]. Our results of the chemotaxis assay showed that both CLEO and LIM significantly inhibited chemotaxis induced by stimulation with fMLP and LTB_4_. However, preincubation of the neutrophils with LIM promoted a more intense inhibition of migration induced by fMLP compared with CLEO. fMLP-induced leukocyte migration involves prostanoid release [31], and the mechanism of action of these substances may be related to the inhibition of cyclooxygenases 1 and 2. Thus, we studied the effects of LIM on the *in vivo* inflammatory response.

Acute inflammation, typically characterized by redness, swelling, pain, and heat, is one of the most important defense mechanisms against invading pathogens [20]. Zymosan, the insoluble polysaccharide component of the cell walls of *Saccharomyces cerevisiae*, is commonly used for the induction of acute peritonitis in mice. In the zymosan-induced inflammatory process, several cytokines, such as tumor necrosis factor (TNF) and interleukin-6 (IL-6), are released. Activation of the complement cascade induces neutrophil accumulation and vascular abnormalities [32, 33]. In BALB/C mice, zymosan-induced peritonitis began with pronounced intraperitoneal plasma exudation associated with increased histamine levels. This was followed by an influx of neutrophils and mononuclear leukocytes, increased levels of plasma/peritoneal fluid chemoattractants (including MCP-1), and the sequential appearance of exudate proinflammatory cytokines (i.e., TNF-*α* followed by IL-1*β* and IL-6) [32]. Essential oil treatments, including* Zingiber officinale* Roscoe, *Rosmarinus officinalis* L., *Cordia verbenacea*, *Pelargonium asperum*, and *Thymus vulgaris* L. essential oils, have been shown to effectively reduce neutrophil chemotaxis [20, 34–37].

**Figure 4:**
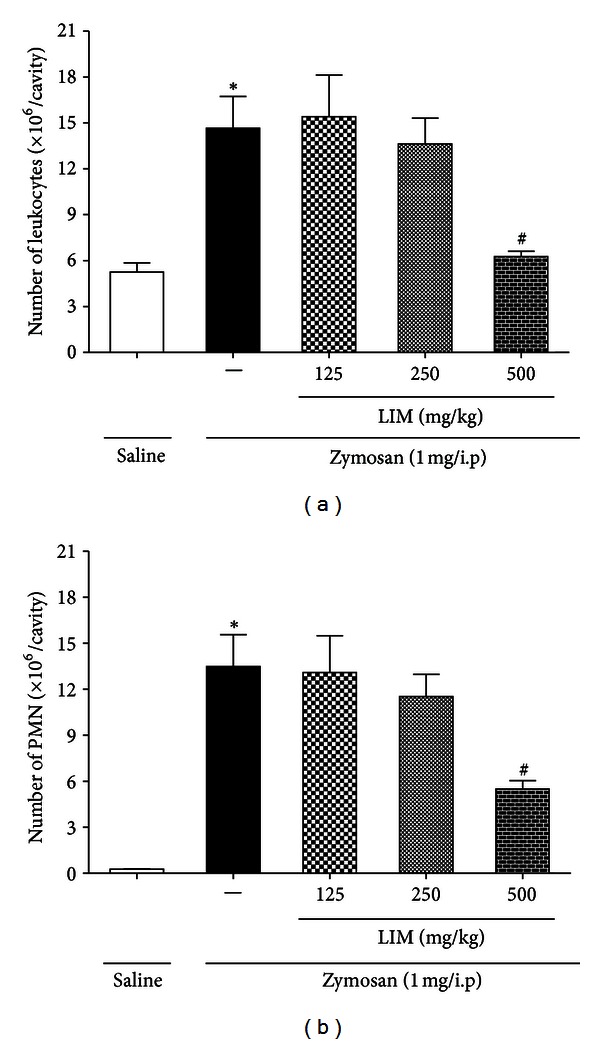
Effect of LIM treatments on leukocyte number. Effect of LIM treatments on leukocyte number 6 hours after zymosan injection (1 mg/cavity/i.p) in Balb/C mice (a) and on PMN number (b). *P < 0.05 versus saline (vehicle). ^#^ *P* < 0.05 compared versus control group. (One-way ANOVA, Tukey's test.)

To evaluate the effects of LIM pretreatment on the migration of inflammatory cells *in vivo*, peritonitis was induced by zymosan. After 6 h of peritonitis induction, an intense inflammatory response was observed, characterized by an increase in the number of peritoneal exudate leukocytes (14.65 ± 2.08 × 10^6^ cells/cavity) compared with the control group (5.25 ± 0.59 × 10^6^ cells/cavity). Many inflammatory mediators are involved in leukocyte migration, such as chemokines, leukotrienes, inflammatory cytokines, and prostaglandins [38, 39]. The animals pretreated with LIM (500 mg/kg) presented a significant reduction of peritoneal exudate leukocyte infiltration compared with untreated animals (Figure 4(a)). The decrease in the number of leukocytes was mainly attributable to a reduction of the number of polymorphonuclear leukocytes (Figure 4(b)). 

**Figure 5:**
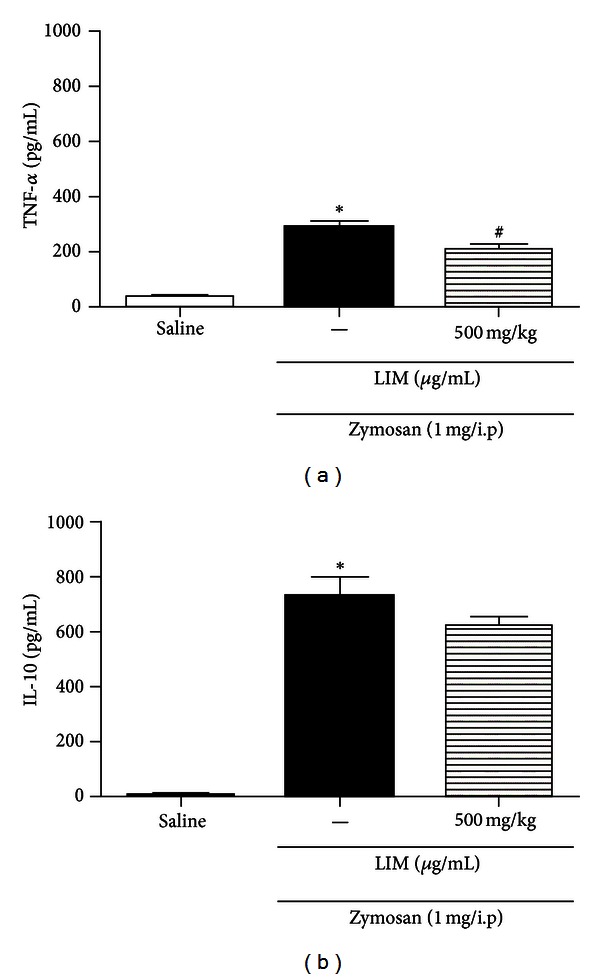
Effect of LIM on levels of TNF-*α* and IL-10. (a) Effect of LIM on levels of TNF-**α** determined in peritoneal exudate, 6 hours after zymosan injection (1 mg/cavity) in Balb/C mice; (b) effect of LIM on levels of IL-10. *P < 0.05 versus saline (vehicle). ^#^ *P* < 0.05 compared versus control group. (Student's *t* test.)

The inflammatory response includes the recruitment of leukocytes and release of inflammatory cytokine, such as TNF-*α*, IL-1, IL-6, IL-10, and others [40, 41]. Various constituents of essential oils effectively inhibit cytokine production. For example, 1,8-cineol inhibited TNF-*α* and IL-1*β* in human lymphocytes. *α*-Humulene reduced TNF-*α* production. Terpinen-4-ol suppressed the production of TNF-*α*, IL-1*β*, IL-8, IL-10, and PGE_2_ by LPS-activated monocytes [11, 42–44]. In the present study, TNF-*α* and IL-10 levels in peritoneal exudate were determined. LIM (500 mg/kg, p.o.) significantly inhibited TNF-*α* levels but not IL-10 levels (Figures 5(a) and 5(b)). Our results indicate that the inhibitory effect of LIM on *in vitro* neutrophil migration may be related to the levels of TNF-*α*, a proinflammatory cytokine. Other studies showed that a *Citrus* essential oil/magnesium salt mixture reduced TNF-*α* levels at the inflammation site. In addition, the levels of the anti-inflammatory cytokine, IL-10, in the citrus oil treatment groups were high compared to those of groups receiving the other treatments. Citrus essential oil itself did not reduce IL-10 levels [45]. LIM exerts anti-inflammatory activity by reducing PGE_2_ production in macrophages [19] and IL-1*α* levels in normal human undifferentiated NCTC 2544 keratinocytes [40]. Other compounds present in CLEO also have some anti-inflammatory effects. For example, linalool inhibits *in vitro* NO formation [12]; *β*-caryophyllene reduces the expression of TNF-*α*, IL-1*β*, interferon-*γ*, and keratinocyte-derived chemokine [10]; *α*-terpineol inhibits the gene expression of the IL-6 receptor [46].

## 4. Conclusions

In conclusion, the present study found that LIM isolated from CLEO had antimigratory activity, likely by inhibiting proinflammatory mediators present in the inflammatory exudate and leukocyte chemotaxis, with the involvement of inflammatory cytokines, such as TNF-*α*. Further studies are needed to elucidate the anti-inflammatory mechanism of these drugs.

## Figures and Tables

**Table 1:** Percentual chemical composition of the *Citrus latifolia *Tanaka essential oil.

Retention time	Compound	Percentual (%)	Identification
8.08	Solvent*	—	MS^a^
8.90	*α*-pinene	2.8	MS, NMR^b^
10.14	**β**-pinene	12.2	MS, NMR
10.59	Solvent*	—	MS
11.54	*p*-cymene	1.8	MS, NMR
11.72	Limonene	62.0	MS, NMR
12.62	**γ**-terpinene	14.2	MS, NMR
13.50	Linalool	0.9	MS, NMR
13.83	Neral	1.6	MS, NMR
15.55	Geranial	0.6	MS, NMR
17.80	—	0.6	No identified
20.40	**α**-terpineol	1.4	MS, NMR
28.29	—	0.6	No identified
24.8	**β**-caryophyllene	1.7	MS

*Chloroform (CHCl_3_).

^a^Mass spectrometry.

^b^Nuclear magnetic resonance.
